# KNOWLEDGE MOBILIZATION TO DEVELOP DEMENTIA-INCLUSIVE COMMUNITIES IN THE DEMSCAPE PROJECT

**DOI:** 10.1093/geroni/igae098.0538

**Published:** 2024-12-31

**Authors:** Habib Chaudhury, Cari Randa-Beaulieu, Kishore Seetharaman

**Affiliations:** Simon Fraser University, Vancouver, British Columbia, Canada; Alzheimer Society of British Columbia, Vancouver, British Columbia, Canada; Simon Fraser University, Vancouver, British Columbia, Canada

## Abstract

Creating a supportive neighborhood built environment that facilitates outdoor mobility, wayfinding, and access to community destinations is a key component in making communities more dementia-inclusive. To help municipalities achieve their vision of dementia-friendly and inclusive streets and outdoor spaces, this Knowledge Mobilization (KM) extension of the Dementia-inclusive Spaces for Community Access, Participation, and Engagement (DemSCAPE) project developed three evidence-based education and training resources for municipal planners, community-based organizations, people with lived experience and care partners. These resources or tools are: 1) “Dementia-Inclusive Planning and Design Guidelines” to inform municipal planners and decision-makers on effective physical planning and design to support people living with dementia; 2) “Neighbourhood Environmental Audit Tool” for community-based dementia advocacy organizations and people with lived experience to conduct audit of the neighbourhood environment and identify intervention projects; and 3) eight-minute documentary video illustrating the lived-experiences of three people living with dementia in urban, suburban and northern areas of British Columbia, Canada. In partnership with the City of Burnaby and the City of Richmond in Metro Vancouver, Canada, three KM activities are being conducted: 1) World Café discussions following screening the short video and an accompanying photo exhibit, 2) focus group discussions with municipal planners to help them apply knowledge of dementia-friendly streets and outdoor spaces to planning and design, and 3) conduct environmental audits of targeted streets in Metro Vancouver engaging people living with dementia and identify actionable built environmental interventions to make their communities more dementia-inclusive.

